# Challenges Facing Women Survivors of Self-Immolation in the Kurdish Regions of Iran: A Qualitative Study

**DOI:** 10.3389/fpsyt.2020.00778

**Published:** 2020-08-14

**Authors:** Javad Yoosefi Lebni, Jaffar Abbas, Farhad Khorami, Bahar Khosravi, Amir Jalali, Arash Ziapour

**Affiliations:** ^1^Health Education and Health Promotion, School of Health, Iran University of Medical Sciences (IUMS), Tehran, Iran; ^2^Antai College of Economics and Management (ACEM), and School of Media and Communication (SMC), Shanghai Jiao Tong University (SJTU), Shanghai, China; ^3^Master of Clinical Psychology, Islamic Azad University, Kermanshah Branch, Kermanshah, Iran; ^4^Master of Women Studies, Shahid Madani University of Azerbaijan, Azerbaijan, Iran; ^5^Substance Abuse Prevention Research Center, Research Institute for Health, Kermanshah University of Medical Sciences, Kermanshah, Iran; ^6^Health Education and Health Promotion, Health Institute, Kermanshah University of Medical Sciences, Kermanshah, Iran

**Keywords:** self-immolation, women, challenges, Kurdistan, qualitative content analysis

## Abstract

**Objectives:**

Women confront many problems after self-immolation, so the purpose of this study was to explore the challenges facing women survivors of self-immolation in the Kurdish Regions of Iran.

**Method:**

This study used a qualitative approach and conventional content analysis. Data were collected through semi-structured interviews with 19 Kurdish women who attempted self-immolation in Iran. They were sampled through purposeful sampling and snowball sampling. The Lincoln and Guba criteria were used to strengthen the research.

**Results:**

The results of data analysis were categorized into five main categories: 1—psychological problems, 2—lack of social and legal supportive structures, 3—incomplete treatment, 4—poor self-care, and 5—social problems. These categories consist of 19 subcategories.

**Conclusion:**

Having been rescued from self-immolation, the women confront many challenges returning to normal life. Reducing these women’s problems and paving the way for their return to life requires multi-dimensional and community-based interventions. Therefore, all social organizations and institutes can cooperate and each of them paves part of the way.

## Introduction

Suicide is one of the oldest social and psychological problems in human societies (1). Among the methods people choose to kill themselves, self-immolation is considered the most dramatic and violent way, which involves victims’ deliberate attempts to use a flammable substance to set themselves on fire (2, 3). The history of self-immolation is long and it is culturally and politically more important than other methods of suicide because it is a method of protesting the social and political structure of society, highly lethal, stigmatizing, and it has serious psycho-social consequences for the survivors and their families (4). Self-immolation rarely occurs in developed countries (5, 6) but it is more prevalent in developing countries such as Iran, Sri Lanka, and India (7). Hanging, drug poisoning, poisoning with pesticides, and self-immolation are some of the most common methods of suicide in Iran. Hanging is more common among men and self-immolation is more common among women (8, 9). Self-immolation accounts for only 1.6% of all burn cases in developed countries (10), while in a country such as Iran, self-immolation is one of the major health problems (11) that is more prevalent among women (12, 13). In Iran, the self-immolation rate is estimated at 4.5 per 100,000 people, accounting for 16% of burn cases treated in hospital, and more than 70% of suicides that lead to deaths were committed by self-immolation (13). Self-immolation is more prevalent in some parts of Iran, especially in the western provinces, which are predominantly Kurdish, and it is one of the most common ways that Kurdish women choose to end their lives (13).

Self-immolation of women in Iran is committed under influence from various factors such as psychological problems, family disputes, and spousal disputes, and social factors such as violence and social protest (1, 14–16). In the Kurdish regions of Iran, self-immolation is also practiced more among women due to imitation and easy access, and can be motivated by protest, intimidating the family and gaining attention, becoming a hero and showing courage, or by instilling guilt in the family and society (13). In recent decades, survivors of severe burn injuries have increased due to advances in medical care and burn care (17). However, people who commit self-immolation and then get rescued experience painful burns that require long-term treatment with social and psychological rehabilitation (1). Burns can have a wide range of physical disorders and emotional and psychological consequences (18). The consequences of self-immolation may be influenced by several factors, including the physical characteristics of the victim, the decision to die, the size of the burn, and the level of hospital facilities (6). Burn treatment is mostly limited to developed countries and it is very expensive and costly. More than 90% of burn cases which result in death occur in developing countries (19). Taking care of patients with severe burns due to self-immolation is always a challenge for nurses and medical personnel of burn wards (20).

Living with wounds from self-immolation can affect a person’s entire life. A study showed that burn injuries cause patients to lose their jobs, as well as requiring them to pay for surgery, psychotherapy, and rehabilitation. They also develop depression and post-traumatic stress disorder (PTSD). In their study, 20 percent of victims of burn injury had PTSD after 2 weeks, and this figure increased to 31.5 percent after 3 months (20). A study in 2012 on the quality of patients’ lives after burn injuries showed that these patients confronted many physical, emotional, and social problems that diminished their quality of life (21). In a qualitative study in 2017 about living with self-immolation wounds among women in Iraqi Kurdistan, it was shown that these women had problems such as feelings of disbelief in God, regret, anger, and hopelessness because of the wounds and living in isolation and solitude (22). Another qualitative study on disfiguring burns and the experienced reactions in Iran found that people with burn deformities received negative reactions from people, causing shame and despair, and their social relationships were hampered (23).

Since care for burn patients has improved considerably over the past 50 years and burn care has shifted from focus on survival to focus on rehabilitation (24), studying the challenges in this area can be of great help to the health community. Much of the research on self-immolation is quantitative (3, 6, 25–27) and few conducted qualitative studies (14, 15, 28) have investigated the factors affecting this phenomenon, and a small number of studies have examined the problems and challenges of women after self-immolation. Carrying out qualitative research in this area can give us useful and comprehensive information and increase our understanding of this phenomenon. Also, gaining up-to-date information from those who have experienced a life with self-immolation wounds can provide useful information to caregivers who work with them to carry out effective interventions for their health improvement and to facilitate the process of their return to normal life. Therefore, this study aimed to explore the challenges facing women survivors of self-immolation in the Kurdish Regions of Iran.

## Material and Methods

### Study Design

The present study used a qualitative approach and conventional content analysis method. One of the important features of qualitative research is that it allows for close attention to be paid to the participants’ point of view and understanding the world through their eyes (29). Qualitative content analysis is an appropriate and coherent method which is used for analyzing text data aimed at better understanding and knowing the phenomenon (30).

#### Sample and Settings

The study participants included 19 women from the Kurdish areas of western and northwestern Iran (including the 4 provinces of Kermanshah, Kurdistan, Ilam, West Azerbaijan) who had been living with self-immolation wounds for the past year. Inclusion criteria included having a history of self-immolation in the past year, having self-immolation wounds, and a willingness to participate in the study (31, 32).

Purposeful sampling method was used to access the samples and in some parts a snowball sampling method was used. The research team tried to make the samples more diverse in terms of demographic characteristics in order to gain a better understanding of the subject (33–37).

### Procedure and Study Materials

A semi-structured interview method was used to collect data. After obtaining permission from Kermanshah University of Medical Sciences, the research team entered the research field. Initially, 12 samples were identified by referring to local health centers, welfare offices, and asking trustworthy locals, and then these 12 people were asked to introduce other women whom they knew had committed self-immolation. Eventually the number of samples reached 19. Interviews were conducted in a quiet environment without presence of other family members. The interview questions were designed by the research team, and before the main interviews began, three pilot interviews were conducted. Pilot interviews showed that the interview questions were appropriate for achieving study goals and general questions of interviews were designed after a little editing. At first some demographic questions were asked to create a friendly and intimate atmosphere, and then the interview started with this general question: “How did you feel and how was your life after you committed self-immolation?” And then other exploring questions were asked like: How did others treat you after the self-immolation? Have you returned to normal life? If no, what made you unable to return to normal life? What problems do you have now?” The average interview time was 40 min. Interviews were carried out in places such as the home, libraries, and parks, and most of the interviews were conducted in the morning when most family members were out. Since the authors of the article were native to the study areas, the interviews were conducted in Kurdish and analyzed in Kurdish, but the quotations in the article were translated by someone who was fluent in both Kurdish and English. During the translation, the first author of the article had continuous monitoring to reach a better translation.

After the first interview, the process of coding and data analysis began and continued until theoretical saturation was achieved through interviewing 19 participants. Theoretical saturation is achieved when new codes or information are no longer obtained by continuing the interviews, and the codes obtained earlier are repeated, so researchers will no longer continue the interviews (38, 39).

### Research Ethics Approval

At the beginning of each meeting, the researchers, introducing themselves and the aim of the study, made sure that the participants’ personal information would be kept confidential and the interview would be stopped if they were unwilling to answer some of questions. Then written and oral consent was obtained to record the interview. The time and location of the interview sessions were determined by the participants and the researchers referred to them at any time that they wished.

### Analysis

In the present study, the method of Graneheim and Lundman was used to analyze the data. This method helps conduct qualitative content analysis methods. It focuses on the analyses of both the manifest or explicit content of the texts and interpretation associated with the texts' latent content (40). At the data preparation stage, the recorded interviews were transcribed and the research team reviewed them a couple of times to gain a general understanding. At the defining semantic units stage, the semantic units were extracted and categorized as compact units. At the coding the text and classifying and developing themes and subthemes stages, the compact units were summarized and labeled with appropriate titles. At the identifying the main themes stage, the sub-categories were grouped based on similarities and differences. In the fifth step, an appropriate title was chosen that could cover the resulting categories.

### Trustworthiness

The Lincoln and Guba criteria including credibility, confirmability, dependability, and transferability were used for the robustness of the research (41). Since three members of the research team were from areas under study and familiar with the culture of these areas, the researchers’ long-term contact with the research field was maintained throughout the research process. The process of data coding and analysis were performed by two members of the research team at the same time. Then all members of the research team, with some researchers acquainted with qualitative research and the cultural and social conditions of the studied areas, reviewed and critiqued the data coding and performed modifications wherever it was necessary. In the end, the categories and subcategories were sent to eight participants to determine whether they expressed their opinions and the situation or not. They confirmed the categories and subcategories. Wherever it was needed, parts of the participant’s responses were quoted.

## Results

The study ended with interviews with 19 women whose demographic characteristics are listed in **Table 1**. The data analysis process resulted in five categories and 19 subcategories (**Table 2**), which are presented below with descriptions and quotes.

**Table 1:** Demographic information of samples.

Variable	Dimension	Frequency	%
Age	16–20	3	15.78
	21–30	9	47.36
	>30	7	36.84
Marriage status	Single	8	42.10
	Married	6	31.57
	Divorced	5	26.31
Residence	Urban	6	31.57
	Rural	11	57.89
	Nomadic	2	10.52
Level of education	Illiterate	5	26.31
	Diploma and lower diploma	11	57.89
	High education (university)	3	15.78
Burned part	Face	11	57.89
	Neck or chest or arm	8	42.10
Occupation	Unemployed	15	78.94
	Self-employed	4	21.05
Self-immolation in relatives?	Yes	17	89.47
	No	2	10.52

**Table 2:** Categories and subcategories.

Categories	Subcategories
Psychological problems	Low self-esteem, fear of the future, feeling guilty and regretful, difficulty adjusting to bodily appearance, lack of emotional support in the family, desire to die, spiritual vacuum
Lack of social and legal supportive structures	Financial penalties for those who attempted self-immolation, lack of financial support for plastic surgery, lack of suitable health facilities
Incomplete treatment	Lack of adequate training for wound treatment, improper behavior of health personnel to wards them, lack of appropriate psychotherapy to rehabilitate them, not involving families in treatment process
Poor cooperation in treatment	Discontinuation of the treatment, disobeying physician’s orders
Social problems	Ostracism, social stigma, disruption of social relationships

### Psychological Problems

Participants had various reasons for this extreme conduct, such as losing appearances, societal and family pressure, and mental disorders.

#### Low Self-Esteem

Most participants usually have low low self-esteem due to their condition.

A 33-year-old woman said, “I have never felt good about myself since I attempted self-burning and harmed myself, and as a result, my face was injured.”

A 16-year-old woman said, “I don’t like myself. I don’t like to look at myself in the mirror at all.”

#### Fear of the Hereafter

Some participants expressed fears about the future since Islamic teachings regard suicide as a major sin.

A 25-year-old woman said, “I lost both this world and the afterworld. I fear the future, if God will punish me for self-immolation.” A 24-year-old woman said, “I am scared of being punished with fire again in the other world, even though I do not like life at all, but I am afraid of dying.”

#### Feeling Guilty and Regretful

Some of them felt guilty after committing self-immolation and felt sorry for having done so.

A 34-year-old woman said, “I regretted it as soon as I burned myself, but it was too late and my neck and body burned before putting the fire out.” A 21-year-old said, “I feel sorry and guilty for burning myself. I have done a big sin. God forgive me.”

#### Difficulty Adjusting to Bodily Appearance

Most participants had a hard time adjusting to their new appearance because of the burn effects on their face and body.

A 36-year-old woman said, “Whenever I see my body, I hate myself. I can’t do anything to get over it.” A 32-year-old woman said, “I used to know myself as the most beautiful girl among my relatives, but now I see myself as the ugliest, I hate myself.”

#### Lack of Emotional Support in the Family

Since suicide in families is usually considered a form of disgrace, girls who commit self-immolation are often blamed and neglected in the family and are not emotionally supported.

A 22-year-old woman said, “Since I committed self-immolation, my family neglects me more than ever before. My family members do not talk with me at all.”

A 24-year-old woman said, “Since I attempted self-immolation, my family started to regard and see me as a criminal. My family believes that I have disgraced them.”

#### Desire to Die

Despite feelings of guilt and remorse, suicidality does not always dissipate after self-immolation. Indeed, some participants expressed death wishes, and one them indicated that she tried to commit suicide on more than one occasion

A 17-year-old said, “After self-immolation, my life became worse than before. I have tried to commit suicide a couple of times, but I don’t do it for my mother’s sake.” A 24-year-old woman said, “Every day I think about death, I’m not pleased with this life at all.”

#### Spiritual Vacuum

Since suicide in Islam is a major sin, some participants were usually unable to spiritually associate with God after being saved and became spiritually vacuumed.

A 22-year-old woman said, “Before I committed self-immolation, I used to talk to God whenever I got very annoyed, but now I feel too shy to talk to God.” A 25-year-old woman said, “Then I feel too shy to pray anymore because of my self-immolation so I feel so bad. I feel like I have no one else”.

### Lack of Social and Legal Supportive Structures

Since suicide is considered a crime under the laws of the Islamic Republic of Iran, people who commit suicide receive the least social and legal support and usually confront more problems for treatment.

#### Financial Penalties

In Iran, those who commit self-immolation face financial punishments and fines. Therefore, many families try to hide self-immolation when they come to the hospital for treatment. However, some hospitals refuse, and some people hide it if they have some social influences. A 28-year-old woman said, “When I was taken to the hospital my family did not tell the doctors that I had committed self-immolation for fear of being fined.”

A 34-year-old woman said, “There is no law in Iran to protect and support women who commit self-immolation. But, there are some laws against them; everyone thinks that you have committed the biggest crime when you commit self-immolation.”

#### Lack of Financial Support for Plastic Surgery

Plastic surgery in Iran costs highly and few people can pay for it. Thus many women with burn problems usually stay the same for the rest of their lives and are not treated. A 22-year-old woman said, “After I was saved from self-immolation, my hand burnt a lot. Everyone said it would be okay, but it did not get normal. They took me to the doctor a few times. It was highly costly, and my family could not afford such massive expenditures. Therefore, it is the same as before. I get annoyed a lot. I have to wear gloves to hide them.” A 17-year-old woman said, “I really want to have plastic surgery on my face, but I don’t have the money to do it. Many times in the morning when I wake up, I put my hand on my face, hoping it may be fine, but I get disturbed and upset when I see it would not heal.”

A 22-year-old woman said, “After I went to the hospital to treat my wounds, I realized that because I committed self-immolation, I couldn’t get any insurance for plastic surgery.”

#### Lack of Suitable Health Facilities

Kurdish areas in Iran are underdeveloped compared to the rest of Iran and have limited access to adequate healthcare facilities. So women who self-immolate often have to travel long distances for treatment, which makes their treatment process difficult. A 16-year-old woman said, “There is no hospital near us. Our roads are so bad that it takes a long time to get to a well-equipped hospital.” A 25-year-old woman said, “If I want to go to the hospital, I have to go to Imam Khomeini Hospital in Kermanshah. We have no one there and it can be very difficult for me to get treatment. I stopped treatment for that reason.”

### Incomplete Treatment

Kurdish women who commit self-immolation are usually not fully cured due to economic and social deficiencies and the prevailing cultural conditions.

#### Lack of Adequate Training in Wound Treatment

Since the participants were generally low educated and health personnel did not have a positive view of them, they usually did not receive adequate training in wound treatment. A 21-year-old woman said, “When I was discharged from the hospital and came home, my wounds got infected, because I really didn’t know how to treat them. The doctors took me and my treatment lightly.” A 34-year-old woman said, “The doctors said I would be fine in a few months if I cared for it well, but they did not say exactly what to do. So my wounds got healed too late and I have their scars left.”

#### Improper Behavior of Health Personnel

Since suicide in Islam is one of the worst acts a person can do, women who commit self-immolation are found guilty and health personnel are usually unkind to them and they may be subject to discrimination. A 24-year-old woman said, “In the hospital, they treated me very badly and didn’t care much about me when they found out I committed self-immolation.” A 34-year-old woman said, “Nurses’ behavior to me was not good at all. They blamed me repeatedly and took little care of me.”

#### Lack of Appropriate Psychotherapy to Rehabilitate Them

There are few counseling centers in the study area; however people do not go to these few centers because of the cultural taboos. So women who commit self-immolation are usually deprived of psychotherapy programs and they cannot get the training needed to return to normal life. A 17-year-old woman said, “As soon as I was discharged from the hospital, no one asked me if my condition improved. I am still scared of fire. Even though they knew I had committed suicide but didn’t have a psychologist to talk with me.” A 24-year-old woman said, “After the self-immolation I have many psychological problems, I couldn’t get over the issue. I was very eager to go to the psychologist but conditions were not met.”

#### Not Involving Families in Treatment Process

Some participants complained that they were not assisted in the treatment process by their families, and most often stated that their family had not received any training on how to deal with them. A 22-year-old woman said, “I had committed self-immolation because of my family, but because they were not told how to treat me later, they continue the same behaviors as before.” A 28-year-old woman said, “My family does not know how to treat me. They want to be sympathetic but they get on my nerves and I get annoyed. I wish they knew how to treat someone who attempted suicide.”

### Poor Cooperation in Treatment

Most participants usually have poor cooperation in treatment after discharge from hospital due to mental and social conditions, so in many cases their wounds do not heal.

#### Discontinuation of the Treatment

Most of the women in the study, due to their mental and social conditions, were reluctant to pursue treatment, so they confronted burn problems for a longer time. A 41-year-old woman said, “After self-immolation, a physician prescribed a couple of physiotherapy sessions for my hand, but I didn’t go.” A 34-year-old woman said, “I don’t really want to get involved in treatment. I got careless.”

#### Disobeying Physician’s Orders

Many of the cases stated that they did not obey the physician’s instructions for treatment and in some cases they self-medicated.

A 22-year-old woman said, “I had no patience to do some of the things my doctor told me to do, and I didn’t do them, sometimes I did the traditional stuff myself, for example, I put honey on my wound.” A 31-year-old woman said, “My doctor told me I had to wear comfortable clothes at home so that my scars would not become infected, but I felt too shy and wore local clothes that caused infection.”

### Social Problems

Most participants usually experience severe social problems after self-immolation due to social and cultural conditions in Kurdish areas that can disrupt their lives.

Ostracism: Because self-immolation is regarded as disobeying God’s commands, women who do so are usually ostracized in society. A 24-year-old woman said, “Everybody looks at me as a sinner. My family says I disgraced them. I don’t like to go out at all.”

A 36-year-old woman said, “Our family and relatives have completely changed their behavior after I committed self-immolation. They didn’t respect me at all. It was good for the first few days but then they didn’t care about me anymore.”

#### Social Stigma

Because burns remain on the hands and faces of these women for a long time, they are easily recognized in the community and this makes many problems for them. A 27-year-old woman said, “At weddings, they all show me to each other and talk about me, nothing is harder than this.” A 41-year-old woman said, “I really want to hide my hand so that no one can see. Because when anyone sees my burn, they focus their attention to me and talk behind me and let themselves think anything about me.”

#### Disruption of Social Relationships

The scars of burns on the body and face of women cause crisis in their lives and severely affect their social relationships. On the other hand, it is difficult for them to get married because of losing their beauty.

A 16-year-old woman said, “I do not like to be in touch with anyone because they all communicate with me out of compassion. I want to be alone more.” A 22-year-old woman said, “With this wound on my face, I don’t think I’ll ever get married”.

## Discussion

This study aimed to explore the challenges facing women survivors of self-immolation in the Kurdish Regions of Iran with a qualitative approach. One of the challenges for women to return to normal life after self-immolation was psychological problems, including low self-confidence, fear of the future, feeling guilty and regretful, difficulty adjusting to bodily appearance, lack of emotional support in the family, desire to die, and spiritual vacuum.

Because of the special circumstances it creates, self-immolation can make many psychological problems for women that can affect their entire lives. Self-immolation wounds can make women not have positive views about their appearance and body and feel less confident. Islam strictly forbids self-harm in any form, and people committing suicide or self-immolation will face punishment in the afterlife (42). However, individuals who survive can seek forgiveness in their life from God. However, if the women survivors repent and regret their evil actions in their lives, God can forgive those people who regret and seek Almighty God’s forgiveness. The doors of forgiveness are always open, and people can directly request forgiveness to God anytime. Women who do so usually cannot communicate well with God afterward. They have some kind of spiritual vacuum that can aggravate their psychological problems.

In previous studies, most victims of self-immolation had psychiatric disorders ranging from 60 to 91% (1). A review study of 27 studies and 582 patients showed that those who attempted self-immolation had emotional, schizophrenic, and personality disorders (11).

Having burn wounds can increase a person’s potentiality for developing complex psychological illnesses (43). Depression, anxiety, and low self-confidence are other common problems among burn survivors (44, 45). Because self-immolation wounds remain on the victim’s body and face and cannot be concealed, the psychological problems that survivors have of self-immolation can be greater than those of other types of suicide. Low self-esteem and difficulty adapting to their new appearance were new findings in the study that have not been studied in previous research on suicide and self-immolation. In the study of Kornhaber et al, shame, regret, and guilt were other experiences that survivors of burn injuries experienced (46). In the studies by Hunter et al. and Cox et al, victims of burn injuries were very concerned about their appearance and also had negative perceptions about their body (45, 47).

Another major challenge that faced the participants when they attempted to return to normal life was the lack of social and legal support structures. Irani people consider self-immolation of self-harm a criminal act and people regard those who attempt suicide as criminals (14). Consequently, people try to hide reporting suicidal attempts at hospitals and patients and their families usually do not report suicide attempts to avoid financial fines and other social problems. Patients will also not receive any funding for rehabilitation if they are diagnosed with self-immolation, and as the costs of rehabilitation are high, most patients will in fact be left out of the treatment process. In addition, burn treatment is costly and requires advanced treatment facilities and equipment (1). Kurdish areas are among the most economically deprived areas in Iran, and suffer from a lack of adequate health care centers. Some patients who have committed self-immolation have suffered a lot from health services due to alack of appropriate treatment centers in the area, and some even gave up on the treatment process. The process of treating burn patients is a long and costly process, and without the financial and non-financial support of the institutions, one cannot expect families to go through the treatment process. In Iran, despite having a very long history of burns, and especially self-immolation, there is no special center to care for these patients and facilitate the process of their return to life (23). In order to reduce women’s problems after self-immolation, by changing the view of considering suicide as a crime and not considering women who have survived self-immolation as criminals, it is possible to provide more support for them. By creating social and rehabilitation institutions for these women the conditions for their easier return to normal life can be provided.

Another category obtained from data was incomplete treatment, which consisted of lack of adequate training in wound treatment, inadequate behavior of health personnel, lack of appropriate psychotherapy programs to rehabilitate individuals, and not involving families in the treatment process.

In fact, most participants were not fully treated. Part of this was due to the lack of appropriate health structures in the areas under study and the greater part was due to cultural and social issues specific to these areas. Women in these areas are mostly illiterate or poorly educated, unable to access internet information sources, etc., and receive insufficient training in wound care at the hospital. Froutan et al. demonstrated that a lack of knowledge and information on burns and their treatment was a main problem experienced by burn patients (48). Also, health and social institutions do not have comprehensive rehabilitation plans for these people, and since their families are often financially poor, they cannot afford private counseling. Women who commit self-immolation are left alone after getting rescued and are likely to face more difficulties than before, so in many cases they attempt suicide again.

Poor cooperation in treatment was another derived category, which consisted of two subcategories of discontinuation of the treatment and disobeying physician’s orders. The treatment of burn injuries is time-consuming and the individual’s role in the treatment process is highlighted, thus the necessity for self-care in burn patients, and especially in self-immolation cases, increases (49). In the research by Litchfield et al. (50), self-management has been identified as one of the factors affecting burn wound healing (50).

The financial costs of self-immolation treatment in Iran are high and most of the cases do not have the financial ability for plastic surgery, so they discontinue treatment and live with burn wounds for the rest of their lives. Also, since the women under study still could not cope with their burns and their rehabilitation processes were incomplete, they had no incentive to continue treatment and had poor self-care and were less likely to follow the physician’s instructions.

Another category was social problems, which included the categories of ostracism, social stigma, and disruption in social relationships. After self-immolation, women are subjected to social pressures that make the process of returning to normal life even more difficult. Self-immolation of women in Kurdish areas is seen as a form of disobedience to God’s commands, so there is a great deal of community pressure on her and her family. The victim’s own family, too, views their daughter’s action as a disgrace and says the victim’s behavior is unforgivable. Thus after the self-immolation, the victim’s situation usually worsens and is usually ostracized in the community. Also, since the self-immolation of women in Kurdish areas is high, having burn wounds quickly creates the impression in the minds of others that a person has attempted self-immolation, so they face a social stigma. It also overshadows social relationships in most cases. And since these women have lost their beauty, in many cases they have low self-confidence and this makes getting married difficult. Disruption of social relationships in patients with burn wounds has also been observed in Rahzani et al. research (23).

The presence of social problems, such as facing social stigma, in people with burn wounds has been widely reported in previous research (51, 52). In Thompson and Kent’s research, fear of not being accepted in society was one of the most common causes of anxiety in people with burn injuries (53). In a study by Mirlashari et al., women who committed self-immolation faced social problems such as ostracism and isolation, and self-immolation wounds affected patients’ social relationships (22).

### Strengths and Limitations

This study was the first to qualitatively examines the problems of women who committed self-immolation in Iran and the Kurdish regions, which can provide useful information for health professionals, policymakers, and government officials to design comprehensive and coherent healthcare projects to support and treat patients in the Kurdish regions of Iran. It will help women survivors of self-burning to return to their normal lives. This study also has some limitations. In some cases, families were hesitant to participate in this research study. The research team educated respondents about the study’s importance and obtained consent of the participants with the support of local trustworthy people. Another limitation of this research study was the lack of a full range of research environment as researchers selected just one person from each province and requested to them to identify the other samples. Some participants were afraid, as they were not familiar with qualitative research methods. They had the fear that their words would be publicly broadcast online. The researchers got their consent by explaining the qualitative method research to them and assured them that this research would not publish their names. Another limitation of this study was that only women who had attempted self-immolation were interviewed, while interviews with the families of these women, as well as caregivers and psychologists working with these women, appeared to provide more comprehensive information. Therefore, it is suggested that in subsequent investigations, the families of the victims, caregivers, and psychologists active in this field be included in the study. It is also suggested that other similar qualitative studies be conducted in other areas of Iran and other countries, especially Islamic countries, in order to better understand women’s problems after self-immolation and to better plan and take action to improve their health.

## Conclusion

Women with self-immolation wounds face many problems in society such as psychological problems, lack of social and legal supportive structures, incomplete treatment, poor cooperation in treatment, and social problems that make the process of returning to normal life difficult and often impossible. Reducing the problems of these women as well as paving the way for a return to normal life requires the social support of various institutions and community-based interventions. Therefore all social institutions and organizations can work together and each of them paves part of the way.

The process of their return to society can be facilitated through developing counseling centers for psychological support and appropriate psychoanalysis to help them adapt to new conditions, training families and involving them in the treatment process for how to properly treat women who commit self-immolation, providing financial support for wound treatment, building equipped hospitals and burn centers in the areas under study to facilitate the treatment process, providing self-care training and enhancing it to accelerate the process of improving patients, carrying out social interventions to eliminate social stigma for those who commit self-immolation, and providing necessary instruction to increase social relationships in the community.

## Data Availability Statement

The datasets analyzed in this study can be obtained from the corresponding author on request.

## Ethics Statement

The studies involving human participants were reviewed and approved by Student Research Committee, Kermanshah University of Medical Sciences. The patients/participants provided their written informed consent to participate in this study. Written informed consent was obtained from the individual(s) for the publication of any potentially identifiable images or data included in this article.

## Author Contributions

All authors contributed to the article and approved the submitted version. JY, AZ, and JA: Conception of study design, data analysis support, interpretation, drafting of article and approved final manuscript as submitted. JY, AZ, and AJ: Data analysis, interpretation, drafting of article, revisions and approved final manuscript as submitted. FK, BK, and AZ: Interpretation, critical review and revisions, approved final manuscript as submitted.

## Conflict of Interest

The authors declare that the research was conducted in the absence of any commercial or financial relationships that could be construed as a potential conflict of interest.
